# A plausible accelerating function of intermediate states in cancer metastasis

**DOI:** 10.1371/journal.pcbi.1007682

**Published:** 2020-03-10

**Authors:** Hanah Goetz, Juan R. Melendez-Alvarez, Luonan Chen, Xiao-Jun Tian

**Affiliations:** 1 School of Biological and Health Systems Engineering, Arizona State University, Tempe, Arizona, United States of America; 2 Key Laboratory of Systems Biology, Center for Excellence in Molecular Cell Science, Shanghai Institute of Biochemistry and Cell Biology, Chinese Academy of Sciences, Shanghai, China; 3 Center for Excellence in Animal Evolution and Genetics, Chinese Academy of Sciences, Kunming, China; University of California Irvine, UNITED STATES

## Abstract

Epithelial-to-mesenchymal transition (EMT) is a fundamental cellular process and plays an essential role in development, tissue regeneration, and cancer metastasis. Interestingly, EMT is not a binary process but instead proceeds with multiple partial intermediate states. However, the functions of these intermediate states are not fully understood. Here, we focus on a general question about how the number of partial EMT states affects cell transformation. First, by fitting a hidden Markov model of EMT with experimental data, we propose a statistical mechanism for EMT in which many unobservable microstates may exist within one of the observable macrostates. Furthermore, we find that increasing the number of intermediate states can accelerate the EMT process and that adding parallel paths or transition layers may accelerate the process even further. Last, a stabilized intermediate state traps cells in one partial EMT state. This work advances our understanding of the dynamics and functions of EMT plasticity during cancer metastasis.

## Author summary

Epithelial-mesenchymal transition (EMT) is a basic biological process, in which epithelial cells undergo multiple biochemical changes, lose cell-cell junctions and polarization, and become a mesenchymal phenotype with migratory and invasive properties. Recent studies have illustrated the existence and importance of the partial EMT states. It has become increasingly apparent that the EMT has strong differentiation plasticity. This plasticity is heavily implicated in cancer cell invasion and metastasis. However, it is still unclear how the number of intermediate states changes the EMT process. Here, we use a hidden Markov model to describe the EMT process. By fitting with the experimental data, we find that unobservable microstates exist within the observable macrostates: epithelial, partial EMT, and mesenchymal. Additionally, we find that increasing the number of states between the start and end of EMT or including alternative transition avenues via parallel paths or transition layers can accelerate the EMT process. This study suggests a non-trivial function of the EMT plasticity during cancer metastasis.

## Introduction

Epithelial-to-mesenchymal transition (EMT) is a fundamental cellular process in which polarized epithelial cells lose various cell-cell junctions and adhesion and gain migratory and invasive properties to become mesenchymal cells [1, 2]. EMT is very important in embryonic development, tumorigenesis, metastasis, tumor stemness, and therapy resistance [3, 4]. Remarkably, EMT is not a binary process but instead proceeds with multiple partial intermediate states, collectively known as partial or hybrid EMT states [3, 5–11]. The partial EMT state retains some characteristics of epithelium but also shows features of mesenchymal cells [12–14]. One partial EMT state was predicted through mathematical modeling of the EMT core regulatory network and was verified with quantitative experiments by our previous works [5, 6]. Thereafter, many different partial EMT states were proposed [8, 9, 15–17]. More and more experimental data shows a different number of partial EMT states in various cancer cell lines [18–23]. Recently, several partial EMT phenotypes were found during cancer metastasis *in vivo* in a skin cancer mouse model [24, 25] and prostate cancer [26]. While many partial EMT states have been found, their functions are still not fully understood during cancer metastasis [4, 27–29].

Currently, the function of partial EMT states has being studied in the context of coupling with other cellular processes. For example, acquisition of stem-like properties dictates its coupling with cancer stemness [11, 30–34], circulating tumor cells (CTCs) [35, 36], and drug resistance [37]. Thus, the partial EMT cells hold the highest metastatic potential. Instead of full EMT, partial EMT is found to be critical for renal fibrosis [38–40]. There are many potential couplings of partial EMT and other biological processes, such as cell cycle [40], renal fibrosis [41] and metabolisms [42]. The cross-talk among the regulators of EMT and other cellular processes guides the coupling mechanism and full functional characteristics of partial EMT states.

While it is important to investigate specific functions of each partial EMT state in the context of additional cellular processes, an interesting question about the general function is whether the number of partial EMT states affect the transition itself. Given that different cell lines may have different partial EMT states, epithelial cells in different systems may also undergo different steps to become mesenchymal cells. Here, we use a general hidden Markov model to describe cell fate transitions during the EMT process. Our analysis makes several non-intuitive predictions. First, several microstates exist within one macrostate, which makes EMT a non-Markov process. Second, increasing the number of intermediate states can accelerate the EMT process. Third, transition in parallel and layer modes can further accelerate the process. Lastly, the existence of a stabilized intermediate state traps cells within their current phenotype. We conclude with discussions on how the dynamics and functions of EMT plasticity are controlled by the number of states during cancer metastasis.

## Materials and methods

### Transition rate and energy barrier

In this work, we assume that the total energy barrier one epithelial cell has to cross to transition to the mesenchymal state is Δ*E*. If there is no intermediate state, then following the Arrhenius equation, the transition rate of EMT is $k=k_{0}e^{-\Delta E/k_{BT}}$, where the value of *k*_0_ is a pre-exponential factor, which determines the time scale of the system. For simplicity, we define the energy unit as *k*_*BT*_, thus we can simplify the equation to *k* = *k*_0_*e*^−Δ*E*^. Suppose there are *N*_*int*_ intermediate states between the epithelial and mesenchymal states and that the energy is divided into *N*_*int*_ + 1 steps; the transition rate for step *i* is $k_{i}=k_{0}e^{-\delta E_{i}}$ where *δE*_*i*_ is the energy barrier for this step and ∑*δE*_*i*_ = Δ*E*. We considered two scenarios:

1. The energy is divided evenly into all the steps, and thus the transition rate for each step is same: $k_{i}=k_{0}e^{-\Delta E/(N_{int}+1)}$.
2. There is one stabilized intermediate state where the energy barrier is *δE*_*s*_ = *mδE*_*i*_. We considered case one: $\delta E_{s}+\sum_{i\neq s}^{}\delta E_{i}=\Delta E$. The transition rate is $k_{i}=k_{0}e^{-\Delta E\frac{1}{\left(N_{int}\;+\;m\right)}}$ for the transition from the regular intermediate states, and $k_{s}=k_{0}e^{-\Delta E\frac{m}{\left(N_{int}\;+\;m\right)}}$ for the transition from the stabilized intermediate state, or case two: the transition rate is $k_{i}=k_{0}e^{-\Delta E\frac{1}{\left(N_{int}\;+\;1\right)}}$ for the transition from the regular intermediate states (same as scenario 1), and $k_{s}=k_{0}e^{-\Delta E\frac{m}{\left(N_{int}\;+\;1\right)}}$ for the transition from the stabilized intermediate state.

### Estimation of *k*_0_ and the energy barrier Δ*E*

The total energy barrier from epithelial to mesenchymal, Δ*E* and *k*_0_, were estimated by the following equations:

$$\begin{array}{c} k_{fit}=k_{0}e^{-\Delta E/(N_{fit}+1)} \end{array}$$

where *N*_*fit*_ = 9 is the number of intermediate states and the transition rate *k*_*fit*_ = 3.4261 is from the best fitting of the experimental data. For each Δ*E*, we can calculate *k*_0_ and the transition rates for all cases.

### Parameter fitting

#### Based on one intermediate state and an irreversible EMT process

First, we considered one intermediate state in the model to fit the parameters. We assumed EMT to be an irreversible process under high dose of inducer TGF-*β*. $\vec{p(t)}=[p_{E}(t),p_{P}(t),p_{M}(t)]$ is the vector that represents the probability of a cell belonging in each state during the process of EMT. Thus, the equation governing the dynamics of $\vec{p}$ is

$$\begin{array}{c} \frac{d}{dt}\vec{p}=[\begin{array}{ccc} -k_{1} & 0 & 0\\ k_{1} & -k_{2} & 0\\ 0 & k_{2} & 0 \end{array}]\vec{p} \end{array}$$

where *k*_1_ is the transition rate from the epithelial state to the pEMT state, and *k*_2_ is the transition rate from the pEMT state to the mesenchymal state.

The equations are solved and the solution is:

$$\begin{array}{c} p_{E}\left(t\right)=e^{-k_{1}t}, \end{array}$$

$$\begin{array}{c} p_{P}\left(t\right)=\frac{k_{1}}{k_{2}-k_{1}}e^{-k_{1}t}-\frac{k_{1}}{k_{2}-k_{1}}e^{-k_{2}t}, \end{array}$$

$$\begin{array}{c} p_{M}\left(t\right)=1-\frac{k_{2}}{k_{2}-k_{1}}e^{-k_{1}t}+\frac{k_{1}}{k_{2}-k_{1}}e^{-k_{2}t}. \end{array}$$

#### Based on a flexible number of intermediate states and an irreversible EMT process

The above model does not accurately describe the experimental data. Thus, we extended the model by considering *N*_*int*_ + 1 microstates; this is based on an assumption that each macrostate, including the epithelial state and pEMT state, consist of multiple microstates. Suppose that EMT is an irreversible process where the cells transition independently from one microstate to the next at the same rate *k*. Based on the assumption that the Δ*E* is evenly divided into these steps, the following equations can be used to represent the dynamics of the probability of the microstates:

$$\begin{array}{c} \frac{d}{dt}\vec{p}=[\begin{array}{ccccccc} -k & 0 & 0 & \ldots & 0 & 0 & 0\\ k & -k & 0 & \ldots & 0 & 0 & 0\\ \vdots & \vdots & \vdots & \ddots & \vdots & \vdots & \vdots \\ 0 & 0 & 0 & \ldots & k & -k & 0\\ 0 & 0 & 0 & \ldots & 0 & k & 0 \end{array}]\vec{p}. \end{array}$$

The solution of this equation is:

$$\begin{array}{c} p_{n}\left(t\right)=\frac{1}{(n-1)!}e^{-kt}k^{n-1}t^{n-1},\left(1\leq n\leq N_{int}+1\right), \end{array}$$

$$\begin{array}{c} p_{M}\left(t\right)=1-\sum_{n=1}^{N_{int}+1}p_{n}\left(t\right). \end{array}$$

#### Based on a flexible number of intermediate states and a reversible EMT process

EMT has some probability to be reversible even under high dose of inducer TGF-*β*, which may result from the noise in the cell or other stochasticity. We also considered a case where the EMT process is reversible with the cells transitioning independently between one microstate and the next at the forward rate *k*_1_ and backward rate *k*_−1_. The following equations can be used to represent the dynamics of the probability of the microstates:

$$\begin{array}{c} \frac{d}{dt}\vec{p}=[\begin{array}{ccccccc} -k_{1} & k_{-1} & 0 & \ldots & 0 & 0 & 0\\ k_{1} & -k_{-1}-k_{1} & k_{-1} & \ldots & 0 & 0 & 0\\ \vdots & \vdots & \vdots & \ddots & \vdots & \vdots & \vdots \\ 0 & 0 & 0 & \ldots & k_{1} & -k_{1}-k_{-1} & k_{-1}\\ 0 & 0 & 0 & \ldots & 0 & k_{1} & -k_{-1} \end{array}]\vec{p}. \end{array}$$

### First arrival time

The first arrival time (FAT) distribution is used to quantify the time cells take to be transitioned into the mesenchymal state or stabilized state in various scenarios. The FAT distribution is $f_{M}\left(t\right)=\frac{d}{dt}p_{M}\left(t\right)$ with normalization ($\int_{0}^{\infty }f_{M}dt=1$).

#### For the one-path scenario

When there is a total of *N*_*int*_ intermediate states, the probability of a cell being in the *n*-th intermediate state is given by:

$$\begin{array}{c} p_{n}\left(t\right)=\frac{1}{(n-1)!}e^{-kt}k^{n-1}t^{n-1},\left(1\leq n\leq N_{int}+1\right), \end{array}$$

$$\begin{array}{c} f_{M}\left(t\right)=\frac{1}{N_{int}!}e^{-kt}k^{N_{int}+1}t^{N_{int}}. \end{array}$$

The corresponding mean first arrival time (MFAT) to the mesenchymal state is

$$\begin{array}{c} \left\langle f_{M}\right\rangle =\int_{0}^{\infty }\left(f_{M}\cdot t\right)dt=\frac{N_{int}+1}{k}=\frac{N_{int}+1}{k_{0}e^{-\Delta E/(N_{int}+1)}} \end{array}$$

which indicates that the MFAT non-linearly depends on *N*_*int*_.

#### For the parallel path scenario

The probability of a cell in the *n*_*i*_-th intermediate state of the *i*-th path is given by:

$$\begin{array}{c} p_{n_{i}}\left(t\right)=\frac{k_{i}^{n_{i}}}{{\left(k_{i}-k_{A}\right)}^{n_{i}}}\cdot (e^{-k_{A}t}-e^{-k_{i}t}\sum_{j=0}^{n_{i}-1}(\frac{{\left(\left(k_{i}-k_{A}\right)t\right)}^{j}}{j!})). \end{array}$$

The FAT distribution is given by:

$$\begin{array}{c} f_{M}\left(t\right)=\frac{dp_{M}}{dt}=\sum_{i=1}^{N_{pth}}k_{i}p_{N_{i}}, \end{array}$$

where *N*_*pth*_ is the number of paths and *N*_*i*_ is the number of intermediate states in the *i*-th path.

The MFAT to the mesenchymal state is

$$\begin{array}{c} \sum \frac{k_{i}+N_{i}\cdot k_{A}}{k_{A}^{2}}, \end{array}$$

where *k*_*A*_ = ∑*k*_*i*_ and *k*_*i*_ is transition rate in the *i*-th path.

#### For the layered scenario

The probability of a cell in the *i*-th layer is given by:

$$\begin{array}{c} p_{L_{i}}\left(t\right)=N_{L1}\cdot (\frac{e^{-N_{L1}kt}}{{\left(1-N_{L1}\right)}^{i}}-e^{-kt}\sum_{j=1}^{i}\frac{{\left(kt\right)}^{i-j}}{\left(i-j\right)!{\left(1-N_{L1}\right)}^{j}}), \end{array}$$

and the FAT distribution is given by:

$$\begin{array}{c} f_{M}\left(t\right)=N_{L1}\cdot k\cdot (\frac{e^{-N_{L1}kt}}{{\left(1-N_{L1}\right)}^{N_{ly}}}-e^{-kt}\sum_{j=1}^{N_{ly}}\frac{{\left(kt\right)}^{N_{ly}-j}}{\left(N_{ly}-j\right)!{\left(1-N_{L1}\right)}^{j}}), \end{array}$$

and the MFAT to the mesenchymal state is given by:

$$\begin{array}{c} \frac{N_{ly}+1/N_{L1}}{k}, \end{array}$$

where *N*_*L*1_ is the number of states in the first layer, *N*_*ly*_ is the number of layers, and *k* is the transition rate from one intermediate state to the next (the same for all steps in the layered case).

### The cases with one stabilized state

For any case that has one stabilized state at *S*, such that 1 < *S* < *N*_*int*_ + 1, the probability of a cell in each state goes as follows:

$$\begin{array}{c} P_{E}\left(t\right)=e^{-k_{1}t}\\ P_{1}=k_{1}te^{-k_{1}t}\\ \vdots \\ P_{S-1}=\frac{k_{1}^{S-1}}{(S-1)!}t^{S-1}e^{-k_{1}t}\\ P_{S}=\frac{k_{1}^{S}}{{\left(k_{1}-k_{2}\right)}^{S}}e^{-k_{2}t}-\sum_{i=1}^{S}\frac{k_{1}^{S}t^{i-1}}{{\left(k_{1}-k_{2}\right)}^{S+1-i}\left(i-1\right)!}e^{-k_{1}t}\\ P_{S+1}=\frac{k_{1}^{S+1}k_{2}}{{\left(k_{1}-k_{2}\right)}^{S+1}}e^{-k_{2}t}-\sum_{i=1}^{S+1}\frac{k_{1}^{S+1}k_{2}t^{i-1}}{{\left(k_{1}-k_{2}\right)}^{S+2-i}\left(i-1\right)!}e^{-k_{1}t}\\ \vdots \\ P_{N}=\frac{k_{1}^{N}k_{2}}{{\left(k_{1}-k_{2}\right)}^{N}}e^{-k_{2}t}-\sum_{i=1}^{N}\frac{k_{1}^{N}k_{2}t^{i-1}}{{\left(k_{1}-k_{2}\right)}^{N+1-i}\left(i-1\right)!}e^{-k_{1}t}\\ P_{M}\left(t\right)=1-(\sum_{i=1}^{N}P_{P_{i}}\left(t\right))-P_{E}\left(t\right). \end{array}$$

Here, *k*_1_ denotes the transition rate from the non-stabilized state and *k*_2_ the transition from the stabilized intermediate state. In order for the state to be stabilized as described, it must be true that *k*_2_ < *k*_1_.

#### The MFAT to the stabilized state

The MFAT to the stabilized state is

$$\begin{array}{c} \frac{S+1}{k_{1}}. \end{array}$$

Thus, the acceleration effect of increasing the number of intermediate states or adding paths or layers also applies to the stabilized state.

#### Mean dwelling time

The mean dwelling time distribution is used to quantify the time cells spend within the stabilized intermediate state for various cases. The ODE of the probability of cells in the stabilized intermediate state is

$$\begin{array}{c} P_{S}^{\prime }=k_{1}P_{S-1}-k_{2}P_{S} \end{array}$$

where *k*_1_*P*_*S*−1_ represents the transition into the stabilized state and *k*_2_*P*_*S*_ represents the transition out. The mean dwelling time (*D*_*S*_(*t*)) is defined as the average time that cells spend within this intermediate state, which only depends on *k*_2_. *D*_*S*_(*t*) is as follows:

$$\begin{array}{c} D_{S}\left(t\right)=\frac{\int_{0}^{\infty }te^{-k_{2}t}}{\int_{0}^{\infty }e^{-k_{2}t}}\;dt=\frac{1}{k_{2}}. \end{array}$$

### Stochastic model

To simulate the stochasticity of the cell state transitions during EMT at the single-cell level, we also developed a stochastic model using the Gillespie algorithm.

1. Define the system according to the cell state transitions during EMT, using the following matrices: *S* is the cell state the transition comes from, *P* is the cell state that the transition is to, and vector *K* denotes the transition rates. For example, for the system with only one path and *N*_*int*_ intermediate states, *S* and *P* have *N*_*int*_ + 2 columns and *N*_*int*_ + 1 rows while *K* has one column and *N*_*int*_ + 1 rows, with the pattern:
  $$\begin{array}{ccccc} S=[\begin{array}{cccccc} 1 & 0 & 0 & \ldots & 0 & 0\\ 0 & 1 & 0 & \ldots & 0 & 0\\ \vdots & \vdots & \vdots & \vdots & \vdots & \vdots \\ 0 & 0 & 0 & \ldots & 1 & 0 \end{array}], & & P=[\begin{array}{cccccc} 0 & 1 & 0 & \ldots & 0 & 0\\ 0 & 0 & 1 & \ldots & 0 & 0\\ \vdots & \vdots & \vdots & \vdots & \vdots & \vdots \\ 0 & 0 & 0 & \ldots & 0 & 1 \end{array}], & & K=[\begin{array}{c} k\\ k\\ \vdots \\ k \end{array}]. \end{array}$$
2. Initialize the system with time *t*_0_ and cell state *x*_0_ = [1; 0; …; 0] as one column vector.
3. Determine the rate of each cell transition: *a* = *Sx*_0_*K*, and *a*_0_ = ∑*a*.
4. Generate two uniformly distributed random numbers, *rn*_1_ and *rn*_2_, between [0 1].
5. Determine the time it takes for the cell state transition: *dt* = *ln*(1/*rn*_1_)/*a*_0_,
6. Determine the step in which the cell state transition occurs, *r*, which satisfies: $\sum_{i=1}^{i=r-1}a(i)<rn_{2}\cdot a_{0}<\sum_{i=1}^{i=r}a(i)$.
7. Update the system: *x*_0_ = *x*_0_ − *S*(*r*, :) + *P*(*r*, :) and *t* = *t* + *dt*.
8. Repeat step 3–7 until *t* is more than the maximum time, *T*_*max*_.

## Results

### EMT is a non-Markov process

**Fig 1:**
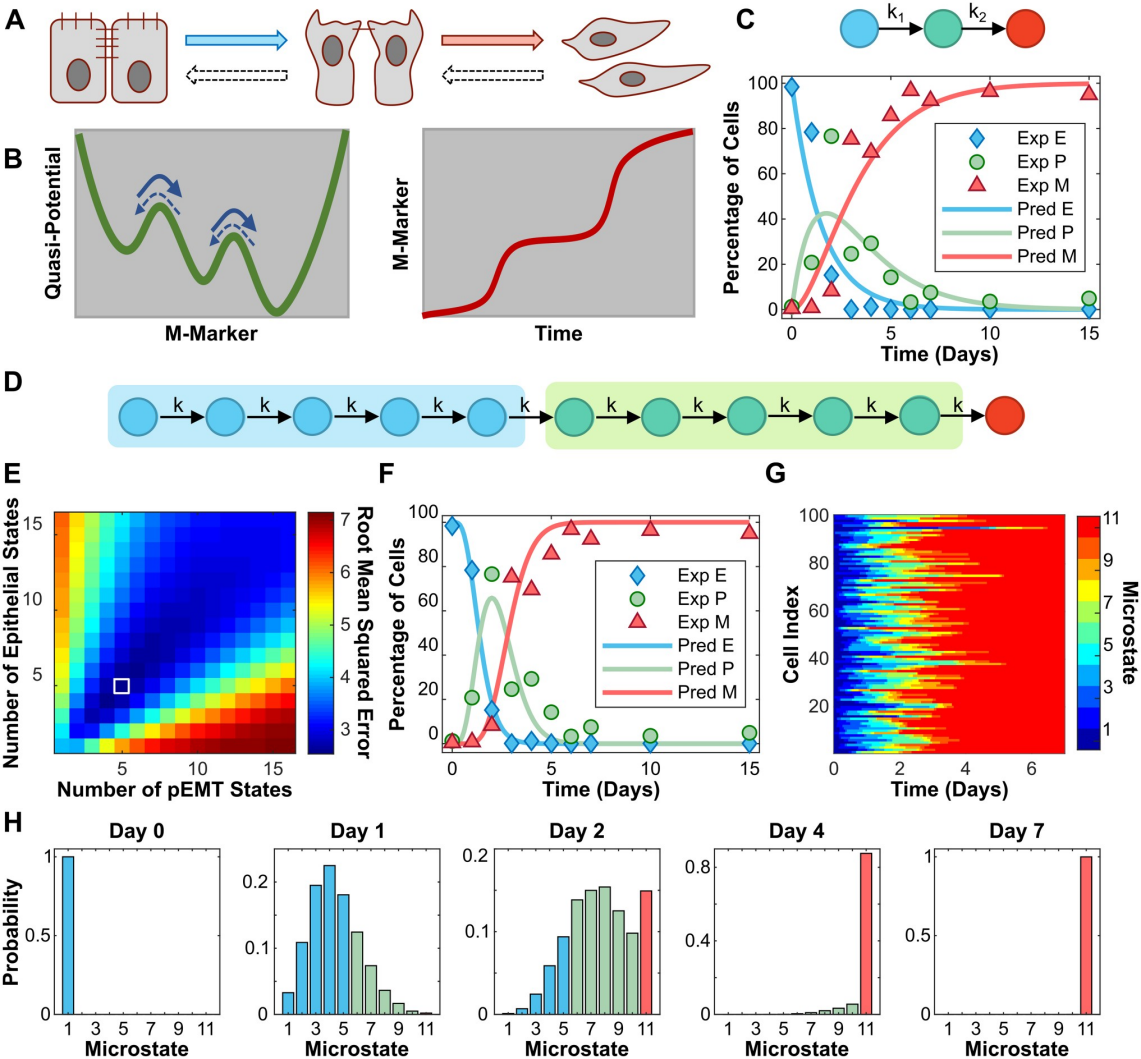
EMT is a non-Markov process. (A) Cell phenotype transitions among epithelial, partial EMT, and mesenchymal states during EMT. (B) Metaphorical three-well landscape in one dimension along the mesenchymal marker (M-Marker) axis shows the cell phenotype transition during EMT. Full arrows show the order of EMT while dashed arrows show reverse process, mesenchymal-to-epithelial transition (MET). (C) Three-state Markov model for EMT (top). Best fit of the three-state model to previous experimental data from Ref. [6] on temporal changes of the percentage of cells in three states during EMT with *k*_1_ = 0.6657, *k*_2_ = 0.4908, and *RMSE* = 13.9 (bottom). (D) An extended model with *N*_*E*_ epithelial microstates (blue circles) and *N*_*P*_ partial EMT microstates (green circles). (E) The fitting score, root mean squared error (*RMSE*), with the extended model in the space of *N*_*E*_ and *N*_*P*_. The best fit is at *N*_*E*_ = 5 and *N*_*P*_ = 5 (white square, *RMSE* = 2.55, *k* = 3.4261). (F) The dynamics of cells in three macrostates during EMT with the best-fitted extended model overlaid with experimental data. (G) Stochastic simulation shows the state transition of 100 simulated cells over time. (H) Probability of cells in each microstate at several time points. 10,000 simulated cells with a stochastic model (see Materials and methods for details) are sampled to calculate the probability.

Consistent with theoretical prediction [5], three steady states were found in TGF-*β* induced EMT in the MCF10A cell line with quantitative measurements of E-cadherin and Vimentin at the single cell level [6]. That is, EMT progresses through three functional cell states in this cell line: from the initial epithelial state to the partial EMT state, where cells lose some of the cell-cell adhesion, and then to the full mesenchymal state (Fig 1A). As shown in Fig 1B (left), this system can be represented with a metaphorical three-well landscape in one dimension along the mesenchymal marker (M-Marker) axis. Each well represents a stable or metastable cell phenotype during the EMT process and the transitions among them are indicated with arrows. With high concentration of TGF-*β*, the cell will be driven from the epithelial state (the first well) to the mesenchymal state (last well) through the partial EMT state (middle well), indicated by solid arrows. It is noted that the energy barrier for the forward transition is much smaller than the energy barrier for the reverse transition of each step at high concentration of TGF-*β*. Thus, the forward transition rate (Fig 1B, solid arrows) is much larger than the reverse transition rate (Fig 1B, dash arrows). The dynamics of the M-Marker (measured by the Vimentin protein) at the single cell level shows two steps with cells staying in the intermediate state temporally (Fig 1B, right) [5]. The underlying mechanism is that during EMT the phenotype changes of one cell are controlled by complex genetic regulatory networks and stepwise activation of multiple feedback loops, as demonstrated by Ref. [5, 6].

Due to the cell-cell variability, the cells do not synchronize the transition through these states but make the transitions in a stochastic manner. However, the fraction of cells in each state shows deterministic dynamics, as seen in the quantified experimental data (Fig 1C) from Ref. [6]. To better understand EMT at the population level, we first build a simple model by assuming that cells are initially held in the epithelial state and will be driven by a high dose of TGF-*β*, as used in the experiments, to the partial EMT state before ending in the mesenchymal state, all with constant average rates (*k*_1_ and *k*_2_) (Fig 1C, top). We fit this simple model with the experimental data and find that the model does not describe the data with strong accuracy (Fig 1C, bottom). An inaccurate fitting was also found using a two-state model without any partial EMT states (S1 Fig, panel A). With three-state or two-state models, the transition from the partial EMT state to the mesenchymal state shows a quicker dynamic than the observed data. Thus, three-state or two-state models are not a good representation of the EMT process at the population level, and the cells still pass through other intermediate states temporarily before reaching the final mesenchymal state, which is the only attractor at high concentration of TGF-*β*.

To find out the underlying mechanism for the observed dynamics, we extend the simple model by adding several microstates for each cell macrostate (Fig 1D) by assuming that some unmeasured variables can further distinguish the epithelial state and partial EMT state into several microstates, with the epithelial and partial EMT states being the macrostates used in describing the overall EMT process. While we do not know the actual number of the epithelial and partial EMT microstates (*N*_*E*_ and *N*_*P*_, respectively), a fitting of the extended model is done with all the combinations of *N*_*E*_ and *N*_*P*_ (Fig 1E) by assuming an equal transition rate from one state to the following state (*k*). According to the fitting score (root mean squared error), the best fit is located when *N*_*E*_ = 5 and *N*_*P*_ = 5 (white square in Fig 1E). The best fitting displayed in Fig 1F shows the optimal conditions in which our model reproduces the EMT dynamics at the population level with better accuracy than that of the assumption of only one microstate in both the epithelial and partial EMT states. It is noted that the three macrostates used in our description are defined by E-Cadherin and Vimentin singe-cell data in Ref. [6], not including other makers. In general, the macrostates are defined by the corresponding steady states observed in the experiment with the defined markers. This implies that the number of macrostates observed may differ depending on the markers used. Four macrostates were defined based on additional makers such as Ovol2 [9]. Here, we limited the fitting of the Markov model to the temporal dynamics of the three macrostates as defined in Ref. [6]. Currently, there is no experimental evidence for the existence of nine intermediate states in this cell line, suggesting the existence of microstates during EMT. These results suggest that EMT is a non-Markov process with many hidden epithelial microstates and partial EMT microstates, which could further be distinguished by measuring other variables in the system.

This conclusion is further confirmed with a stochastic model to demonstrate the evolution of cell transitions in these microstates (see Materials and methods for more details). Fig 1G shows 100 typical simulated stochastic trajectories of cells to complete the EMT process. With all cells beginning in the epithelial state, prominent cell-to-cell variation is shown in the time it takes a cell to convert to different microstates, indicating unsynchronized transition at the single cell level. At the population level, the statistic results with 10,000 simulated cells show the dynamics of the distribution of cells in the microstates (Fig 1H). Few reach the mesenchymal state (red bar) by day two. However, most cells will reach the final EMT state by day four. This is also consistent with the experimental data [6] (S1 Fig, panel B). Furthermore, fitting the experimental data with a model by considering the reversible state transitions suggests more microstates (S2 Fig, panel A). Taken together, our analysis based on fitting a general Markov model with the experimental data suggests that EMT is a non-Markov process, where several microstates exist within the epithelial and partial EMT states.

### Increasing the number of intermediate states can accelerate EMT

**Fig 2:**
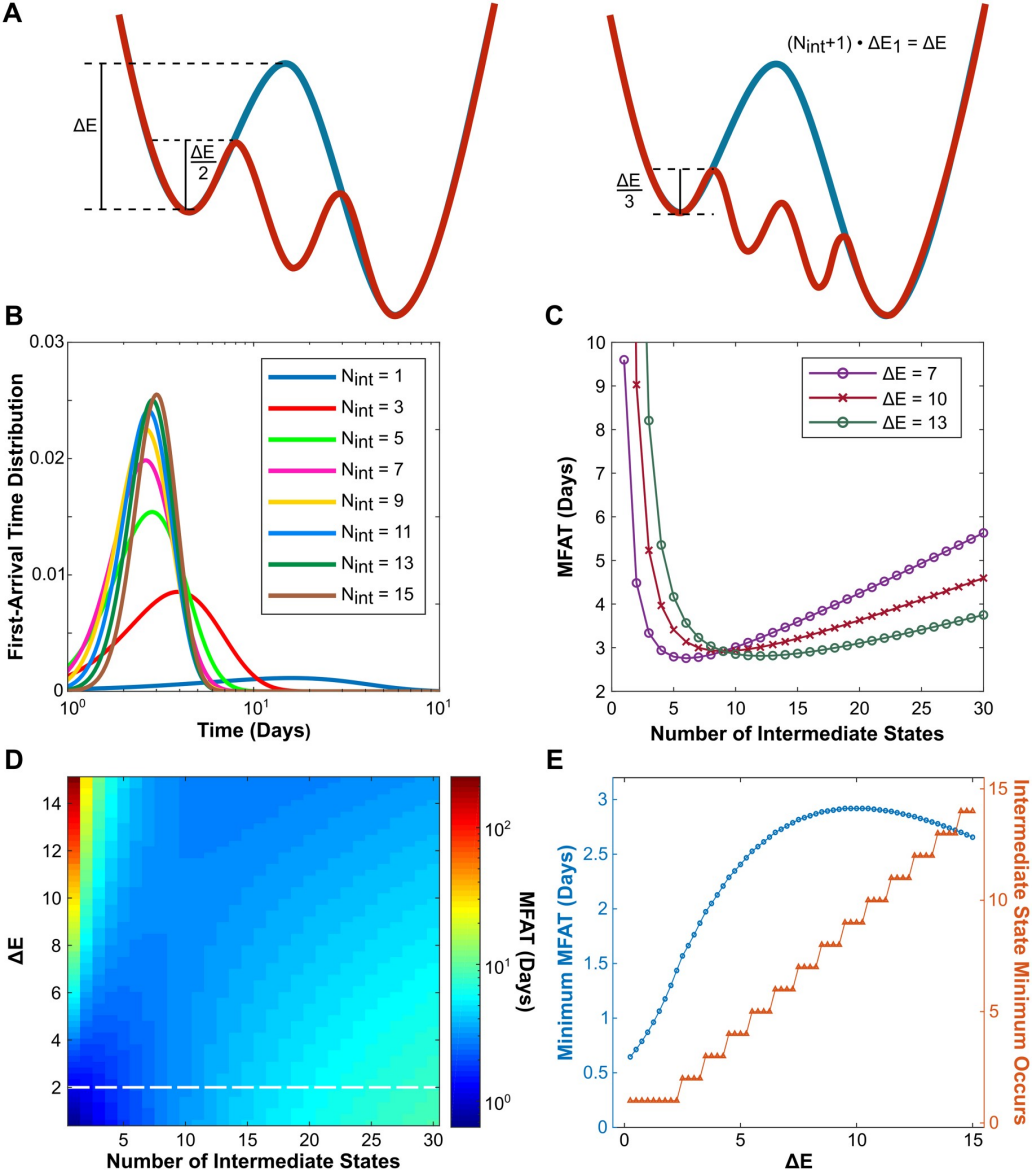
Increasing the number of intermediate states can accelerate the EMT process. (A) The assumptions that the total energy barrier (Δ*E*) for the full transition from epithelial to mesenchymal is fixed to compare the effects of the number of intermediate states (*N*_*int*_) on the dynamics of EMT. The energy barrier is Δ*E*/(*N*_*int*_ + 1) for each step for the system with *N*_*int*_ intermediate states. (B) The distribution of the first arrival time to the mesenchymal state with different *N*_*int*_ and fixed Δ*E* = 10. (C) The dependence of the mean first arrival time (MFAT) on *N*_*int*_ for three different energy barrier values as indicated. (D) MFAT in the space of Δ*E* and *N*_*int*_. A dashed white line represents the threshold above which MFAT shows a non-monotonic dependence on *N*_*int*_. (E) The dependence of the minimum MFAT (blue) on Δ*E* alongside the intermediate state that this minimum occurs (red).

Our analysis with a Markov model for EMT at the population level based on the experimental data of MCF10A cells suggests that multiple microstates exist in each macrostate during the EMT process. However, the number of microstates and macrostates can be cell-type specific. Different numbers of observable steady states have been found during EMT and cancer metastasis [9, 15–23], which leads us to wonder how the number of micro- and macro-states affects the EMT dynamics and cancer metastasis. For this reason, we now consider all states between the initial epithelial and final mesenchymal as “intermediate” states in order to generalize our results and allow for application into cancer cell lines. To compare the systems with different numbers of intermediate states, we made one assumption: the total energy barrier for the full transition from epithelial (E) to mesenchymal (M) is the same despite the total number of states needed to complete EMT. As shown in Fig 2A (left), if the energy barrier from E to M is Δ*E* without any intermediate states (blue curve), adding one additional state makes the transition two steps and the barrier becomes Δ*E*/2 (red curve) for each step. Similarly, the barrier is Δ*E*/3 for each step if two intermediate states are considered (Fig 2A, right, red curve). This assumption comes from the hypothesized monotonical energy gradient based on epigenetic changes between different EMT states [43] and existence of ‘checkpoints’ in the EMT continuum [44]. This assumption is also consistent with the cascading bistable switches mechanism in which EMT proceeds through step-wise activation of multiple feedback loops [5, 6, 9, 45]. The cells need to change the profiles of gene expression to make the full EMT transition. Under this assumption with one intermediate state, the cells can make changes on the expression of some genes as the first step for a partial transition, and then make changes on the rest of the genes for the second step to complete the transition. The profiles of the genes that control the eukaryotic cell phenotypes are usually sophisticated by positive feedback loops to avoid undesired random phenotype switching from noises or short pulse stimulus. These feedback loops can be mutual inhibitions between two groups, one of them controls cell phenotype I while the other group controls cell phenotype II. Thus, the cell fate transitions through EMT intermediate states are analogous to crossing a series of energy barriers controlled by these positive feedback loops.

First, we systematically study how the EMT process changes with fixed Δ*E* and the number of intermediate states, *N*_*int*_. The distributions of the first-arrival time (FAT), defined as the time that cells take to arrive to the mesenchymal state, is used to measure how fast the EMT process is completed. As shown in Fig 2B, with increase of *N*_*int*_, the FAT distribution first shifts to the left and then to the right, becoming increasingly more narrow. To see this affect more clearly, we further calculate the mean first arrival time (MFAT) and find a non-monotonic dependence of MFAT on *N*_*int*_ (Fig 2C). As *N*_*int*_ increases, the MFAT first shows a rapid decrease. However, after a certain *N*_*int*_, the MFAT begins to slowly increase again. These data suggest that the partial intermediate states have a potential function of accelerating the EMT process. Furthermore, we analyze how Δ*E* affects the EMT process. As shown in Fig 2C, the MFAT displays the same non-monotonic pattern for three different Δ*E* values, although the position of the minimum varies. At these minimum points, the EMT process is completed the fastest. This interesting phenomena is further confirmed by the analysis of the MFAT in the space of Δ*E* and *N*_*int*_ (Fig 2D). The same trend of MFAT can be observed for all Δ*E* > 2 (threshold shown by the dash line). This result suggests that the EMT process is not a continuous process but proceeds with a finite number of discrete microstates.

For a Δ*E* value lower than the threshold, MFAT constantly increases with *N*_*int*_ (Fig 2D). The distribution of the first-arrival time for one case with small Δ*E* is shown in S3 Fig. One can see that the transition time is already very small in this case and that increasing intermediate states barely increases the transition rate for each step, thus causing more time to complete full EMT. This is generally not the case for the EMT process in a real system, as the minimal MFAT is less than one day, but it demonstrates the underlying mechanism of the non-monotonic dependence of MFAT on *N*_*int*_. That is, increasing *N*_*int*_ after the minimum does not help to increase the transition rate for each step, it just simply increases the number of steps. Thus, there is a trade-off to accelerate EMT through increasing the number of intermediate states. The dependence of the minimum MFAT alongside the intermediate state in which it occurs ($N_{int}^{min}$) is shown in Fig 2E. With increase of Δ*E*, both the minimal MFAT and $N_{int}^{min}$ increase, although a small decline of minimal MFAT is observed for large Δ*E*. Taken together, a potential function of multiple partial EMT states is to accelerate EMT, and the number of intermediate states necessary to achieve fastest EMT increases with the total energy barrier.

### Adding parallel paths or transition layers further accelerates the EMT process

**Fig 3:**
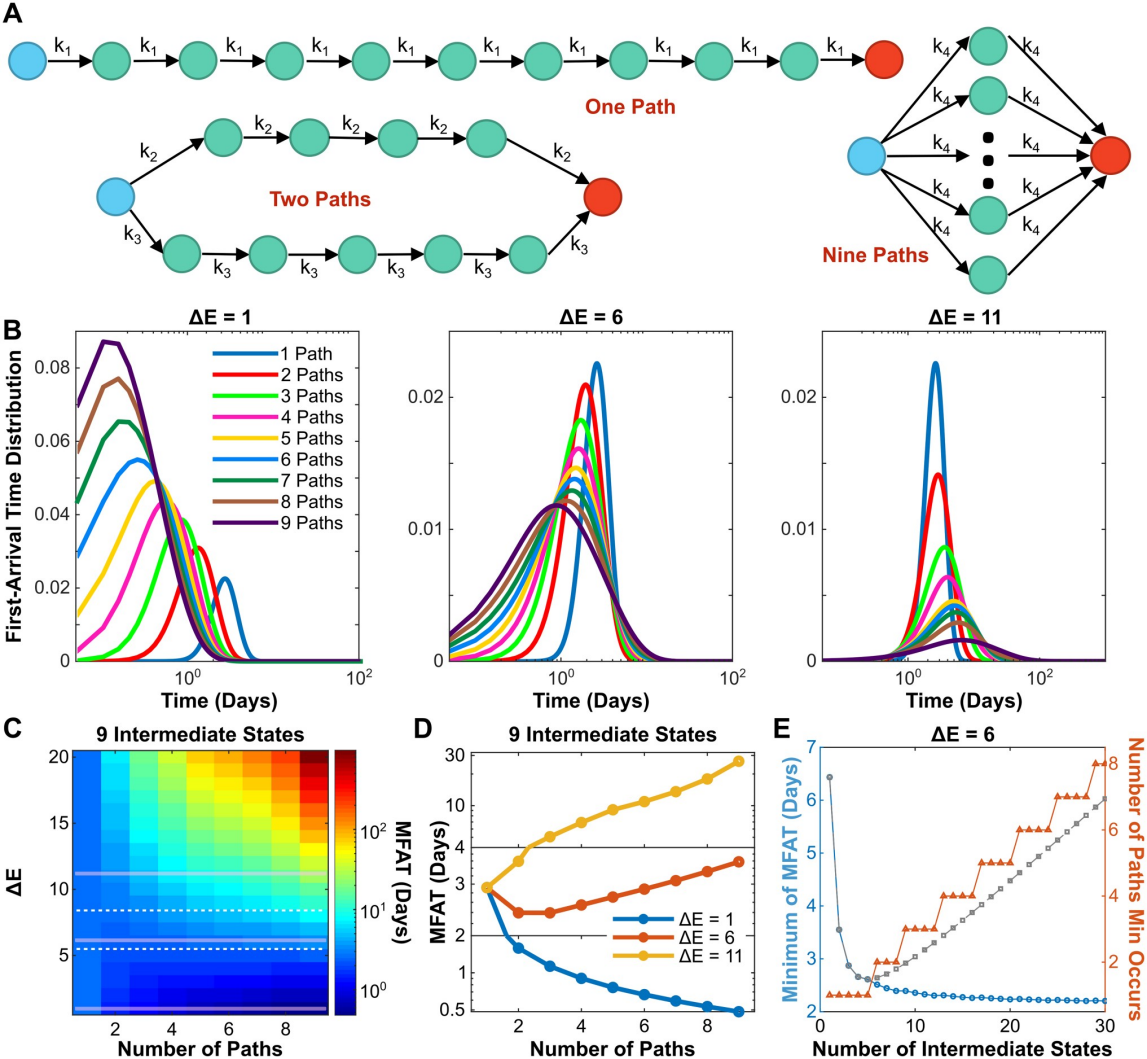
Adding parallel paths can further accelerate the EMT process. (A) Diagram of cell phenotype transition through nine intermediate states with multiple parallel paths. (B) Dependence of FAT distribution on *N*_*pth*_ with three different Δ*E* values and *N*_*int*_ = 9. (C) Phase diagrams showing MFAT subjected to *N*_*pth*_ and Δ*E* with *N*_*int*_ = 9. Dashed lines represent two thresholds for monotonic and non-monotonic dependence of MFAT on *N*_*pth*_. (D) Three typical examples show the monotonic and non-monotonic dependence of MFAT on *N*_*pth*_ according to Δ*E* value. (E) The dependence of minimal MFAT (blue curve) with a comparison to one parallel path (grey dashed line), and the *N*_*pth*_ the minimum occurs on (red) for Δ*E* = 6.

We have discussed the scenario with only one path in which epithelial cells proceed through a step-wise transition of intermediate states to become mesenchymal cells. Recently, parallel EMT paths were reported [46, 47]. Here, we consider multiple paths in parallel (Fig 3A) and study how the number of paths (*N*_*pth*_) affects EMT. For *N*_*int*_ intermediate states, there are *N*_*int*_ possible cases (*N*_*pth*_ = 1 ∼ *N*_*int*_) based on the one simplification that each path contains close to the same number of intermediate states. For example, if there are nine intermediate states and two paths, four states would belong to one path and five to the other (Fig 3A).

To demonstrate the effect of multiple paths, we first study the system with nine intermediate states (*N*_*int*_ = 9, the best fit from Fig 1E). The FAT distribution to the mesenchymal state is shown in Fig 3B under various *N*_*pth*_ and three levels of energy barrier, Δ*E*. For small Δ*E* (left panel), the FAT distribution shifts to the left as *N*_*pth*_ increases, implying that more paths create a faster transition. However, for large Δ*E* (right panel), the FAT distribution reflects the opposite, implying that more paths create a slower transition. These also suggests a non-monotonic dependence of FAT on *N*_*pth*_ for moderate Δ*E*. As shown in Fig 3B (middle panel), the FAT distribution peak shifts to the left but the distribution width becomes larger with increase of *N*_*pth*_. This suggests that the monotonic/non-monotonic relationship between the FAT and *N*_*pth*_ is dependent on the energy barrier.

To further confirm this conclusion, we systematically study how the MFAT of the mesenchymal state changes in the space of Δ*E* and *N*_*pth*_ (Fig 3C). The MFAT dependence on *N*_*pth*_ indeed shows three trends according to the value of Δ*E*. For small (below the bottom white dashed line) or large (above the top white dashed line) values of Δ*E*, the dependence is monotonic, either decreasing or increasing respectively as exemplified in Fig 3D (blue and yellow curves). On the other hand, for a bounded range of Δ*E* values between the two white dashed lines (Fig 3C), it is non-monotonic as exemplified in Fig 3D (red curve). The non-monotonic dependence of MFAT on *N*_*int*_ can change depending on *N*_*pth*_ (S4 Fig). For *N*_*pth*_ = 1, MFAT shows a non-monotonic dependence on *N*_*int*_, the same as Fig 2C. However, the position of the minimum shifts to the right as *N*_*pth*_ is increased to 2∼3. The non-monotonicity is then lost with a continuous increase of *N*_*pth*_. The minimum of MFAT decreases monotonically with the increase of *N*_*int*_. In order to maintain this dynamics, the number of parallel paths must also increase with *N*_*int*_ (Fig 3E). That is, increasing *N*_*pth*_ makes the dependence of MFAT on *N*_*int*_ more monotonically decreasing. These data suggest that increasing both *N*_*int*_ and *N*_*pth*_ together accelerates the EMT process.

**Fig 4:**
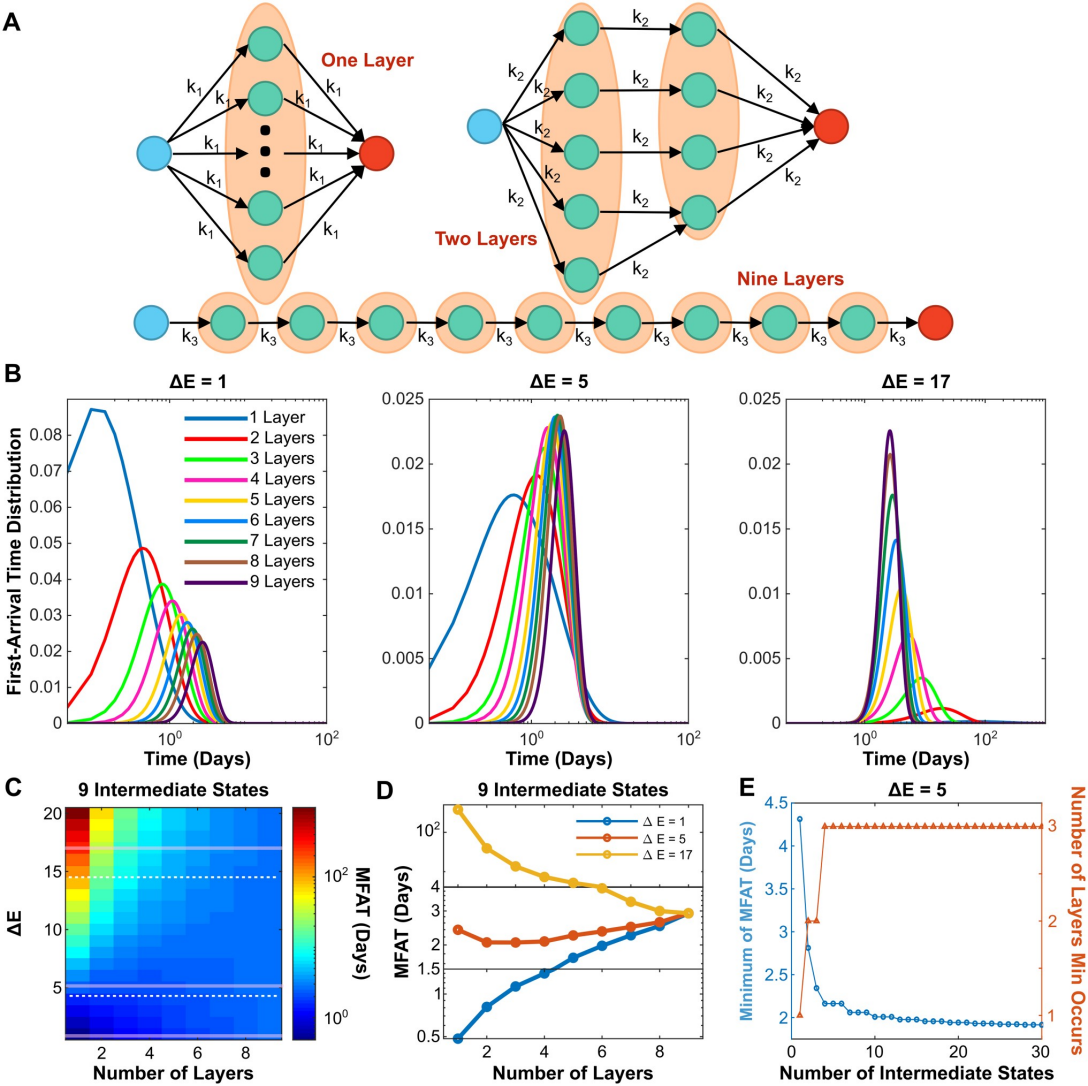
Adding transition layers can further accelerate the EMT process. (A) Diagram of cell state transition through nine intermediate states with multiple transition layers. (B) Dependence of FAT distribution on the number of layer *N*_*ly*_ with three different Δ*E* and *N*_*int*_ = 9. (C) Phase diagrams showing MFAT subjected to *N*_*ly*_ and Δ*E* with *N*_*int*_ = 9. Dashed lines represent two thresholds for monotonic and non-monotonic dependence of MFAT on *N*_*ly*_. (D) Three typical examples show monotonic and non-monotonic dependence of MFAT on *N*_*ly*_ according to Δ*E* value. (E) The dependence of minimum MFAT (blue curve) and the *N*_*ly*_ the minimum occurs on (red) for Δ*E* = 5.

We have now discussed the scenario in which there is only one path and the scenario of multiple paths in parallel and found that increasing the number of the intermediate states or parallel paths could accelerate the EMT process. Here, another scenario is considered by assuming that the epithelial cell has to pass a fixed number of intermediate states to become mesenchymal. In other words, the epithelial cell must pass *N*_*ly*_ of layers to become mesenchymal (Fig 4A). This is similar to the parallel paths scenario but has one difference; it is possible that two paths converge at one intermediate state in the layered scenario. The difference between two topologies is exemplified in S5 Fig, panel A. One can see that a single extra transition step exists in the layered case compared with the parallel case. In the layered topology, the transition rates in all steps are the same, which depends on the number of layers *N*_*ly*_. The MFAT to the mesenchymal state as a function of Δ*E* is not equivalent for the two cases (S5 Fig, panel B). Interestingly, the two curves cross at one specific Δ*E*. For Δ*E* values below this cross-point, EMT is completed faster in the parallel topology. However, for higher Δ*E* values, the parallel topology completes EMT at a slower rate than the layered topology.

Similar to the parallel path scenario, the first-arrival time distribution depends on Δ*E* (Fig 4B). If Δ*E* is small, the FAT distribution shifts to the right with increase of *N*_*ly*_ (Fig 4B, left panel), while it shifts to the left as Δ*E* becomes large (Fig 4B, right panel). The FAT shows a non-monotonic dependence on *N*_*ly*_ with the moderate Δ*E* (Fig 4B, middle panel). Fig 4C shows the MFAT in the space of *N*_*ly*_ and Δ*E* for the system with nine intermediate states. In general, a higher energy barrier gives a slower mean time arrival regardless of the number of layers. However, the trend of the MFAT versus *N*_*ly*_ depends on Δ*E*. MFAT increases with *N*_*ly*_ for small Δ*E* (below the bottom white dashed line), but decreases for large Δ*E* (above the top white dashed line) and shows a non-monotonic dependence for moderate Δ*E* (between the two white dashed lines), as exemplified by the three cases in Fig 4D. Similar to the parallel path scenario, the minimum of MFAT decreases monotonically with *N*_*int*_ as long as more layers are provided for the system with large number of *N*_*int*_ (Fig 4E). This is also confirmed with various values of Δ*E* (Fig 4E and S6 Fig).

Taken together, adding more parallel paths or transition layers can further accelerate the EMT process. In both scenarios, a non-monotonic dependence of the mean first arrival time on the number of paths or layers is found. The MFAT increases with the number of intermediate states after the minimal if the system only has one path, but can further decrease with multiple paths or transition layers. Thus, a combination of increasing the number of intermediate states and parallel paths (or transition layers) can always accelerate the EMT process.

### Stabilized intermediate state traps a cell within its current phenotype

**Fig 5:**
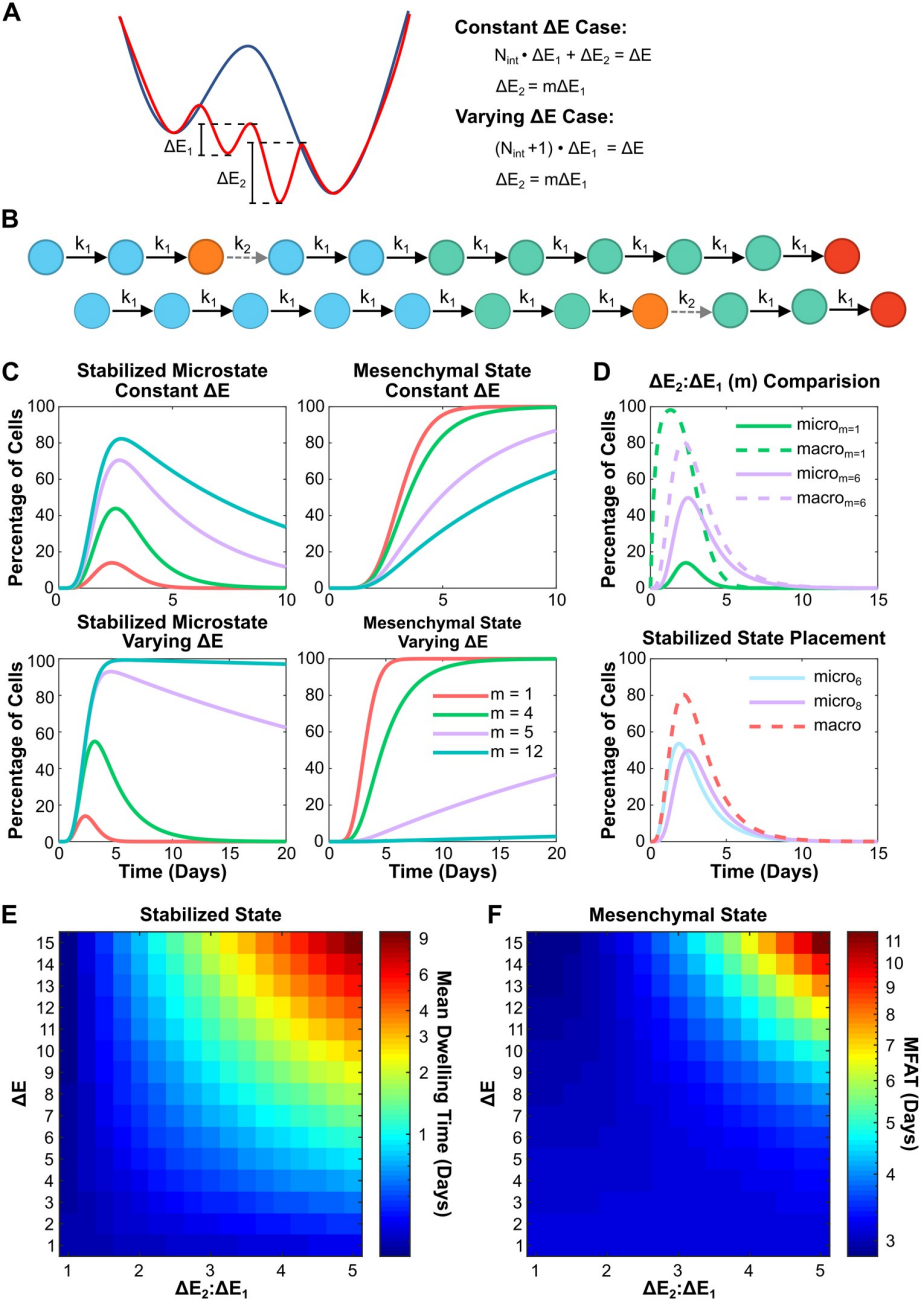
A stabilized intermediate state traps a cell within its current phenotype. (A) The metaphorical landscape of EMT with one stabilized state, which has a deeper well (Δ*E*_2_) than the others (Δ*E*_1_). Two cases were considered here: (1) the constant Δ*E* case in which Δ*E*_2_ increases and Δ*E*_1_ decreases but overall energy barrier Δ*E* remains the same, and (2) the varying Δ*E* case in which only Δ*E*_2_ increases and Δ*E*_1_ remains the same. (B) Example of a stablized state occurring within the EMT process. Orange represents the stabilized intermediate state and the gray dashed arrow represents the transition from this stabilized state (*k*_2_). (C) The distribution of cells at the stabilized state and the mesenchymal state under different energy barrier ratio, *m* (= Δ*E*_2_: Δ*E*_1_). (D) Top: The distribution of the cell at the stabilized microstate (solid lines, the 6th microstate) or at the corresponding macrostate (dash lines, including the 6-10th microstates) with *m* = 1 (green, non-stabilized) or *m* = 6 (purple, stabilized). Bottom: The distribution of the cell at the stabilized microstate (solid lines) or at the corresponding macrostate (red dashed line) with the stabilized state in 6th intermediate state (blue) or the 8th intermediate state (purple). (E-F) Phase diagrams showing mean dwelling time at the stabilized state (E) and MFAT to mesenchymal (F) subjected energy barrier ratio and the total energy barrier Δ*E*, in the constant Δ*E* case.

We have discussed the scenarios in which the same energy barrier is considered for each step during the EMT process. However, it is very possible that one of the intermediate states is stabilized by specific regulators for some cancer cells [48], making the transition from this step more difficult. While mesenchymal cells contribute to cancer metastasis in many cases, the partial EMT states were found to be more aggressive [30, 49]. Here, we consider this scenario where one of the intermediate states is more stable than the others; we call this state the “stabilized” state, which is related to certain states within the more aggressive forms of cancer cells. As shown in Fig 5A, our metaphoric landscape now has one well that is deeper than the others. In this stabilized state, more energy is needed for the transition to the next state. That is, the energy barrier for the transition at this step (Δ*E*_2_) is larger than the energy barrier for other steps (Δ*E*_1_). Two cases are considered here: 1) Δ*E*_2_ increases and Δ*E*_1_ decreases in order to maintain the overall energy barrier Δ*E* (Fig 5A, “constant Δ*E* case”), and 2) Δ*E*_2_ increases as Δ*E*_1_ remains the same to change the overall energy barrier (Fig 5A, “varying Δ*E* case”). Fig 5B represents the diagram of the EMT process with one stabilized state, which includes two transition rates, *k*_1_ as the transition rate from the non-stabilized states, and *k*_2_ as the transition from the stabilized state.

Similar to the mechanism for the accelerated transition to the mesenchymal state in Figs 2–4, increasing the number of intermediate states before the stabilized state and adding layers or paths should also accelerate the transition to the stabilized state, given that the formula of the MFAT to the stabilized state is similar to the one to the mesenchymal state (see Materials and methods). To further understand the impact of a stabilized state on EMT, the distribution of cells at the stabilized state and the mesenchymal state is analyzed with different Δ*E*_2_: Δ*E*_1_ ratios. As the ratio increases, the dwelling time inside the stabilized state grows and it takes more time to arrive to the mesenchymal state for both the constant and varying Δ*E* cases (Fig 5C). That is, the greater the ratio, the more time the cancer cells can stay in the aggressive stabilized state for cancer metastasis. It also implies that cells stay longer in the macrostate where the stabilized intermediate state belongs (Fig 5D, top panel) and that the placement of the stabilized intermediate state within this macrostate does not affect the macrostate behavior (Fig 5D, bottom panel). The dependence of the mean dwelling time within the stabilized state and the MFAT is further analyzed. As shown in Fig 5E and 5F, with increase of either the ratio Δ*E*_2_: Δ*E*_1_ or the overall energy barrier, the mean dwelling time within the stabilized state and the MFAT increase. The same dependence is found for the varying Δ*E* case (S7 Fig, panels A-B) but the scale of changes significantly increases. Another difference between two cases is the reverse dependence of MFAT on Δ*E*_2_: Δ*E*_1_ as shown in S7 Fig panels C-D when Δ*E* is small. Taken together, a greater overall energy barrier or greater intermediate state energy barrier ratio will cause for a longer stay in the aggressive stabilized state as well as a slower transition into the mesenchymal state; this may contribute to cancer metastasis.

## Discussion

Multiple partial EMT states have been proposed and verified to exist in the EMT process, and the number of states involved depends on the cell line. Here, we focused on the dynamics and functions of EMT plasticity and provided microstate and macrostate concepts for EMT. Based on the parameter fitting of a Markov model of EMT with the experimental data, we proposed a statistical mechanism for EMT in which many unobservable microstates exist within the observable macrostates. That is, EMT is a process with many microstates, which can be mapped into different observable macrostates. The microstates may encompass other dimensions of the cell that are coupled to the EMT process, such as the cell cycle, stemness, and metabolism. How the number of microstates is determined is not clear but is believed to largely depend on the cell line, which may be revealed with analysis of single-cell time-course data [50].

Here, we performed a systematical analysis on how the number of intermediate states changes cell transformation. We considered several scenarios including a single transition path, parallel transition paths, and layered transitions. We found that in the single transition path scenario, increasing the number of intermediate states can accelerate EMT, but too many intermediate states can also potentially decelerate EMT, thus showing non-monotonic behavior. However, adding parallel paths or transition layers can further accelerate the EMT process, especially for a system with a large number of intermediate states.

Our results suggest that the number of intermediate EMT states may function as an indicator for the malignancy of the cancer. That is, the more intermediate EMT states in one cancer type, the better the chance it will metastasize. A complementary feature of multiple intermediate phenotypes was reported to stabilize the cancer stem cell population [51]. Thus, the malignancy level can be quantified by the number of potential intermediate EMT states. It is estimated that metastasis causes 90% of deaths related to cancer [49]. If we can stop or slow down the transition, we have a great chance of treating cancer. Our results suggest one potential strategy targeting on the EMT spectrum, i.e., reducing the number of the potential partial EMT states.

Lastly, the existence of a stabilized state within the EMT process causes a slower transition. It is reported that cells in the partial EMT state contribute more to cancer metastasis than the mesenchymal state [52]. Further more, some partial EMT states can be stabilized by the cancer cells [53]. Our simulation shows that stabilizing one intermediate state will trap the cells there for a much longer time period, slowing down full EMT. Multiple intermediate states emerge due to various interlinked positive feedback loops formed in the regulatory network of EMT, which has been demonstrated in many previous works that combine mathematical modeling and quantitative experiments [5–11, 45, 54, 60]. It also has been shown that removal of counteracting determinants traps cells in the rare myeloid transition state [55]. Similarly, if we can tune the strength of the feedback loops that are responsible for the generation of these partial EMT states, the partial EMT states will destabilize and even disband. This will make the first step of metastasis mediated by EMT or partial EMT more difficult and thus give us more time to treat the cancer.

Here in this work, we did not consider the molecular mechanism of the intermediate states but focused on the how the number of the intermediate states affect the EMT process. Further work is needed to verify the prediction derived here and the detailed molecular mechanism needs to be determined for the potential therapeutic targets. Our results suggest that a small perturbation of the signaling pathway could change the EMT process significantly and thus EMT-related diseases, such as cancer metastasis and renal fibrosis. Our previous work on renal fibrosis suggests that knockout of the EMT genes might not be the optimal treatment design for acute kidney injury and long-term fibrosis [41]. Thus, future works can search control strategies to slow down or accelerate EMT with dynamic perturbation of the signaling pathway toward optimal treatment of cancer or fibrosis.

The accelerating function of intermediate states on EMT is analogous to the phenomena in protein folding [56]. It is becoming increasingly apparent that many other cellular processes have multiple intermediate states instead of just one binary or continuous process. For example, stem cell differentiation consists of many intermediate states [57–59]. Thus, it will be exciting to analyze one biological process in different perspectives, including statistical mechanics, mathematical modeling, and single-cell analysis.

## Supporting information

S1 Fig Effects from change in the number of states during EMT. (A) Best fit of the two-state model to previous experimental data from Ref. [6] on temporal changes of the percentage of cells in two states during EMT with *k* = 0.2855 and *RMSE* = 16.67. (B) The dynamics of the EMT process with the stochastic model overlaid with experimental data from Ref. [6]. The simulation data is sampled with 10,000 cells from the stochastic model.
(TIFF)

S2 Fig Fitting the experimental data with a model that considers reversible cell state transitions suggests more microstates during EMT. (A) EMT progression through a continuum of reversible intermediate states. (B) The fitting score, root mean squared error (*RMSE*), for the model with reversibility at the space of *N*_*E*_ and *N*_*P*_ gives the best fit at *N*_*E*_ = 9 and *N*_*P*_ = 8 (white square, *RMSE* = 0.0064, *k*_1_ = 7.1258, and *k*_−1_ = 0.6694). (C) The dynamics of the EMT process with the best-fitted model of reversible intermediate states overlaid with experimental data from Ref. [6].
(TIFF)

S3 Fig FAT distribution for different number of states during EMT. The FAT distribution to the mesenchymal state with different numbers of intermediate states and Δ*E* = 0.1.
(TIF)

S4 Fig Adding parallel paths changes the dependence of MFAT to the mesenchymal state on N_int_. MFAT as a function of *N*_*int*_ under various *N*_*pth*_ with Δ*E* = 6.
(TIFF)

S5 Fig Comparison of the path-dependent and layer-dependent topologies. (A) Diagram of cell phenotype transition through fifteen intermediate states with four parallel paths (left) and four transition layers (right). (B) The dependence of MFAT on Δ*E* under three similar path- and layer-dependent topologies.
(TIFF)

S6 Fig Adding transition layers changes the dependence of MFAT to the mesenchymal state on N_int_. Minimum MFAT (A) and the corresponding number of layers (B) in the space of number of *N*_*int*_, and Δ*E*.
(TIFF)

S7 Fig Comparison of two mechanisms of stabilizing one intermediate state on EMT dynamics. (A-B) Phase diagram of the mean dwelling time of the stabilized state (A) and the MFAT to the mesenchymal state (B) on the energy barrier ratio, Δ*E*_2_: Δ*E*_1_, and total energy barrier, Δ*E*, for the varying Δ*E* case. (C-D) Phase diagram of the MFAT to the mesenchymal state on energy barrier ratio Δ*E*_2_: Δ*E*_1_ and total energy barrier Δ*E* (≤ 1) in the constant Δ*E* case (C) and the varying Δ*E* case (D).
(TIFF)
