# Supplementary figures and images for: A plausible accelerating function of intermediate states in cancer metastasis
Source: PLoS Comput Biol. 2020 Mar 10;16(3):e1007682. doi: 10.1371/journal.pcbi.1007682 (PMC7083331; doi:10.1371/journal.pcbi.1007682)

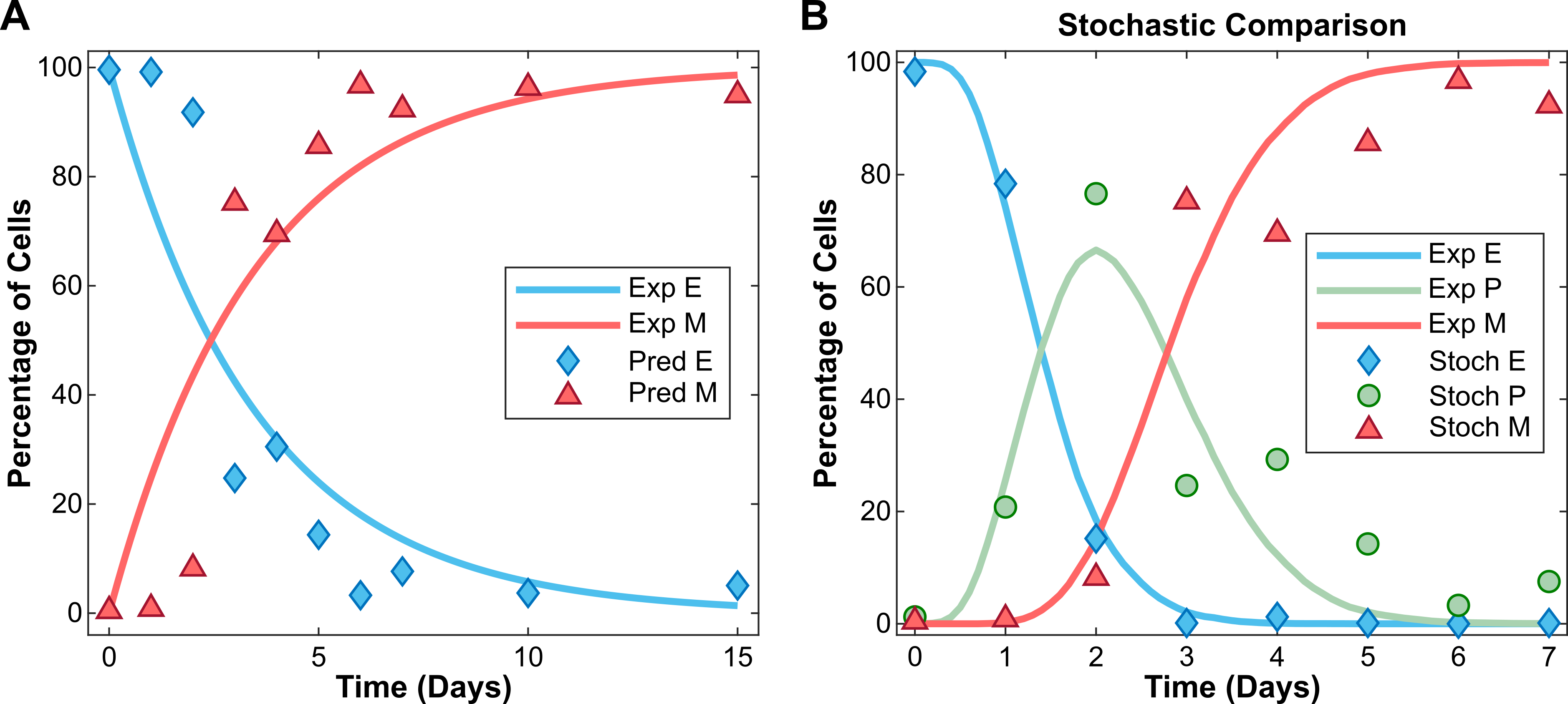

Supplement: S1 Fig — (A) Best fit of the two-state model to previous experimental data from Ref. [6] on temporal changes of the percentage of cells in two states during EMT with k = 0.2855 and RMSE = 16.67. (B) The dynamics of the EMT process with the stochastic model overlaid with experimental data from Ref. [6]. The simulation data is sampled with 10,000 cells from the stochastic model. (TIFF) [file pcbi.1007682.s001.tiff]

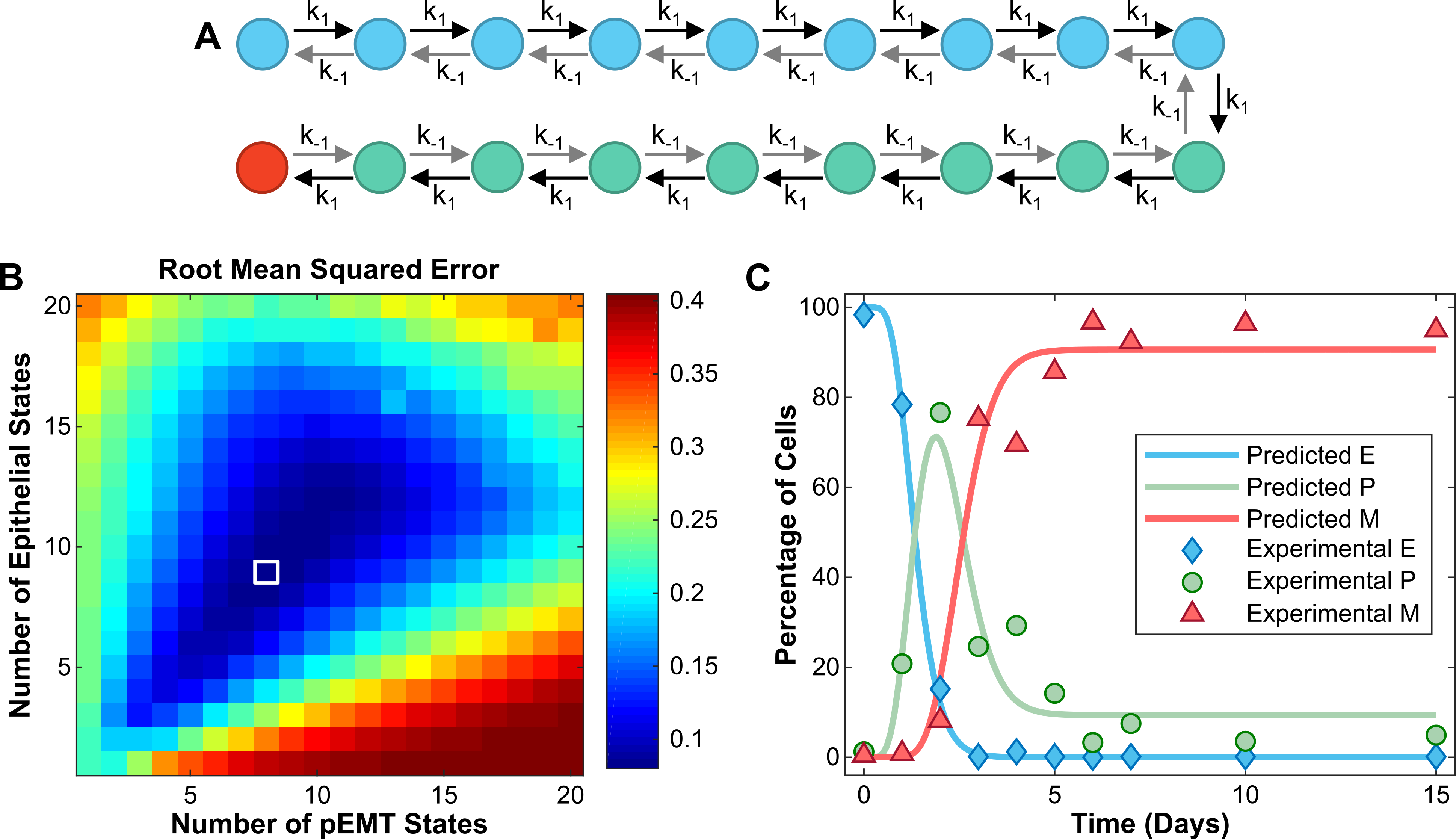

Supplement: S2 Fig — (A) EMT progression through a continuum of reversible intermediate states. (B) The fitting score, root mean squared error (RMSE), for the model with reversibility at the space of NE and NP gives the best fit at NE = 9 and NP = 8 (white square, RMSE = 0.0064, k1 = 7.1258, and k−1 = 0.6694). (C) The dynamics of the EMT process with the best-fitted model of reversible intermediate states overlaid with experimental data from Ref. [6]. (TIFF) [file pcbi.1007682.s002.tiff]

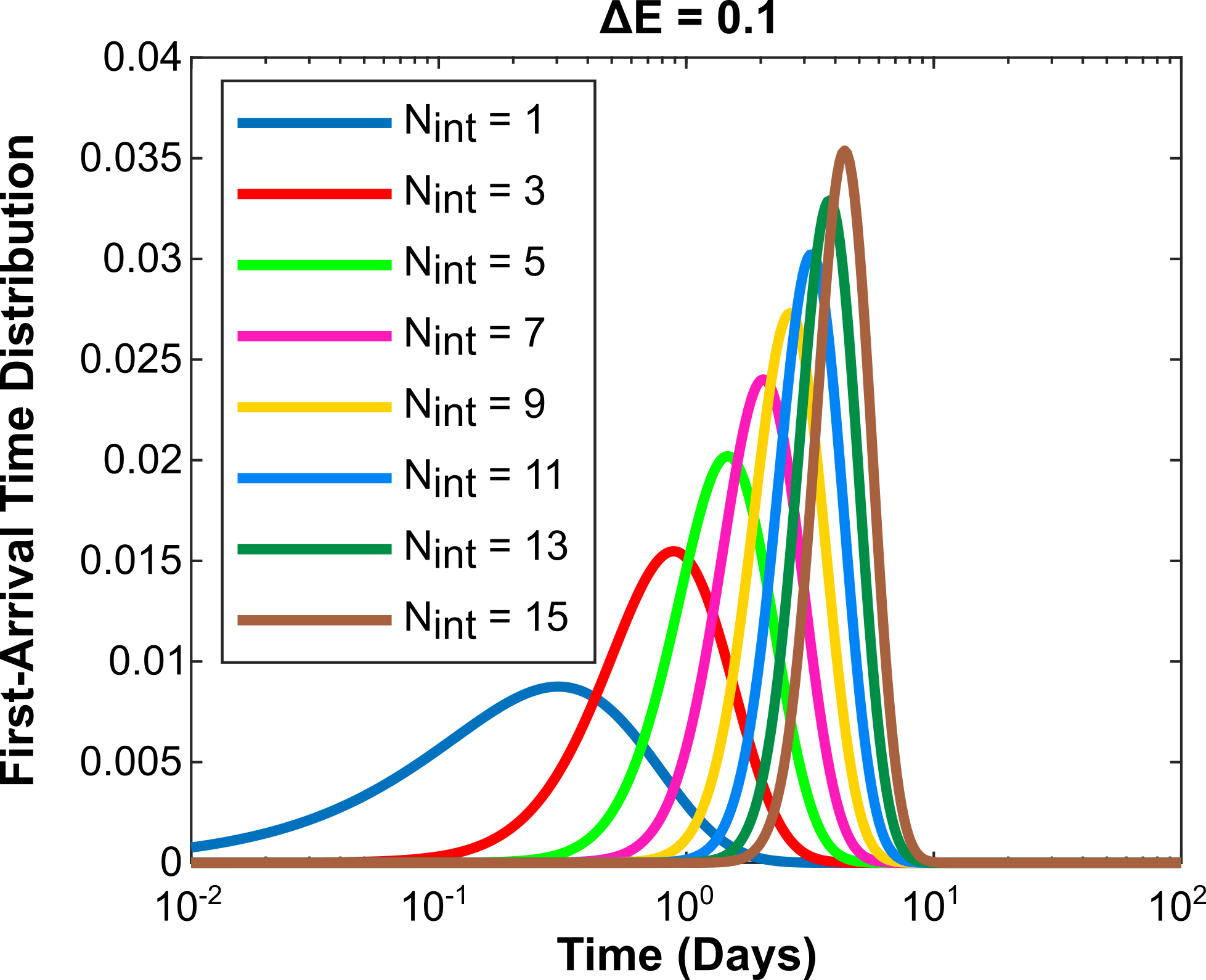

Supplement: S3 Fig — The FAT distribution to the mesenchymal state with different numbers of intermediate states and ΔE = 0.1. (TIF) [file pcbi.1007682.s003.tif]

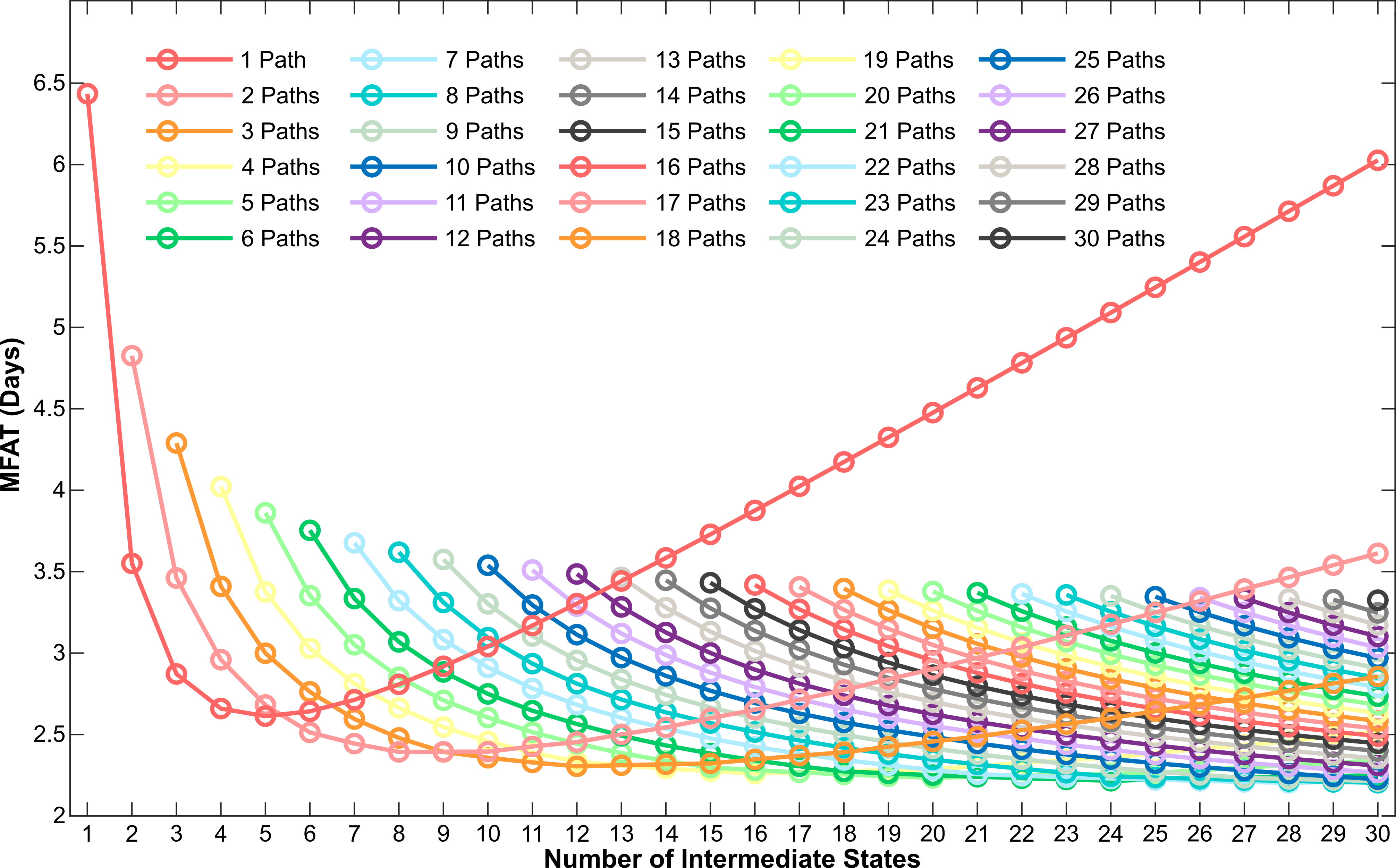

Supplement: S4 Fig — MFAT as a function of Nint under various Npth with ΔE = 6. (TIFF) [file pcbi.1007682.s004.tiff]

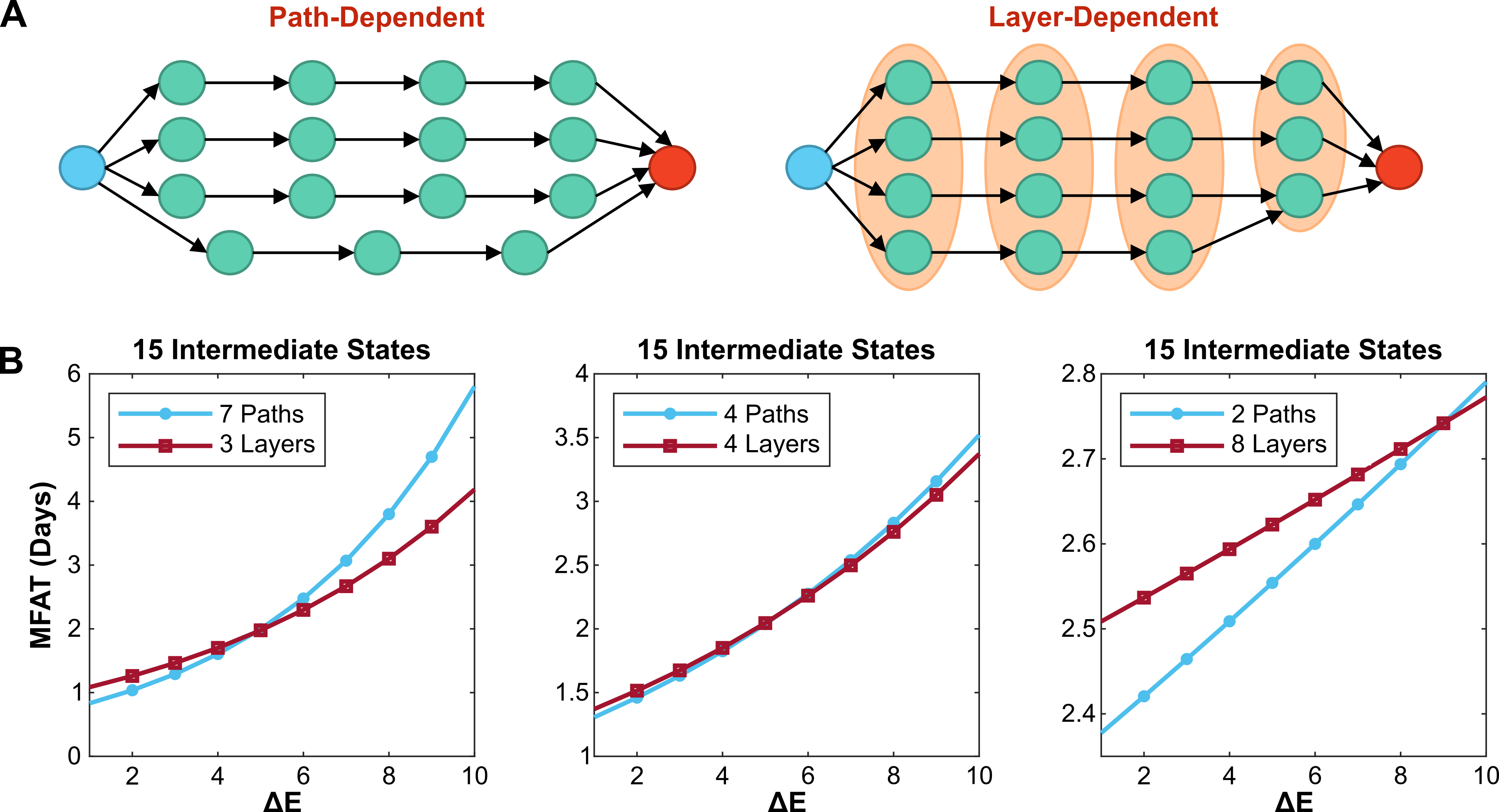

Supplement: S5 Fig — (A) Diagram of cell phenotype transition through fifteen intermediate states with four parallel paths (left) and four transition layers (right). (B) The dependence of MFAT on ΔE under three similar path- and layer-dependent topologies. (TIFF) [file pcbi.1007682.s005.tiff]

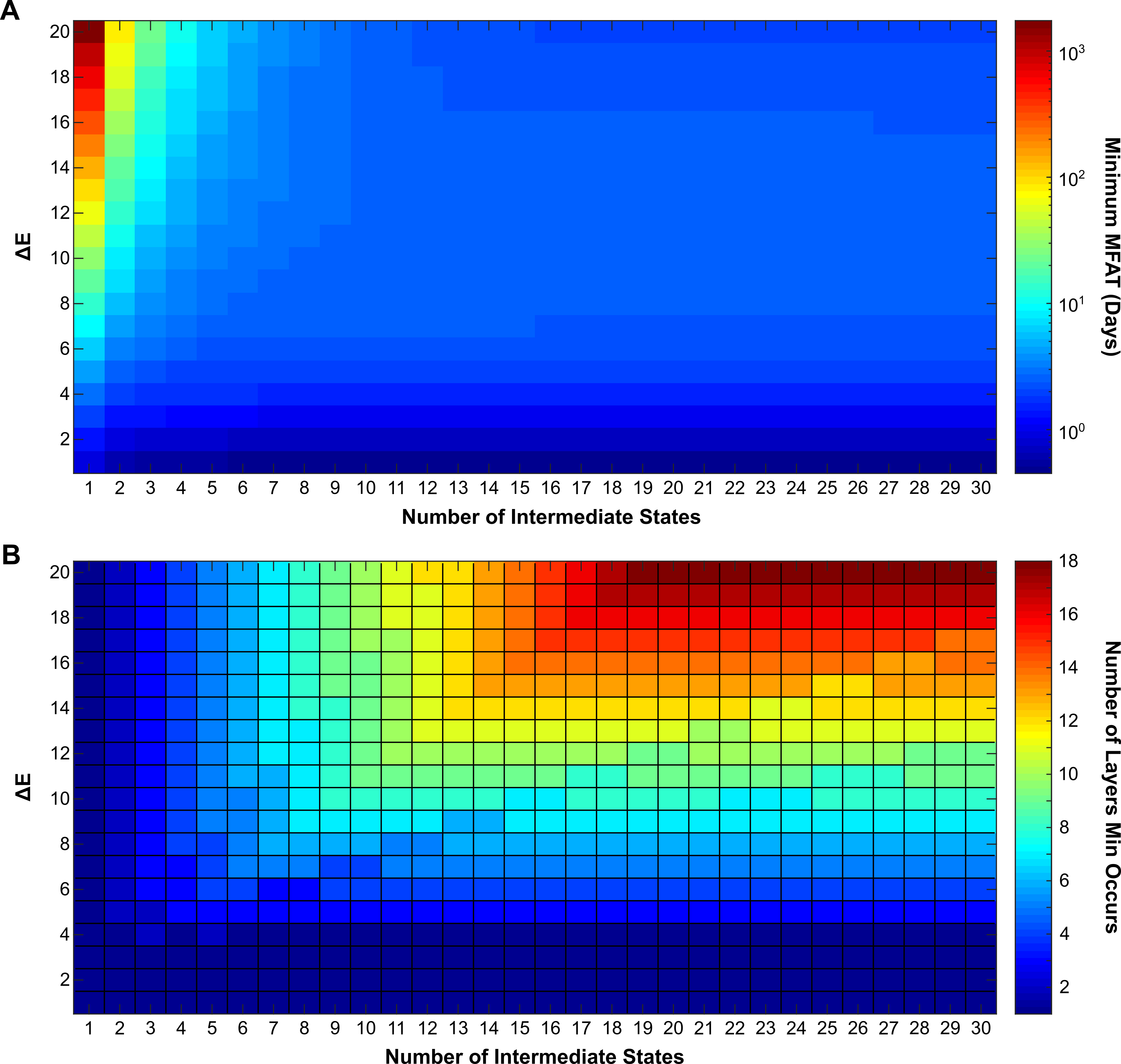

Supplement: S6 Fig — Minimum MFAT (A) and the corresponding number of layers (B) in the space of number of Nint, and ΔE. (TIFF) [file pcbi.1007682.s006.tiff]

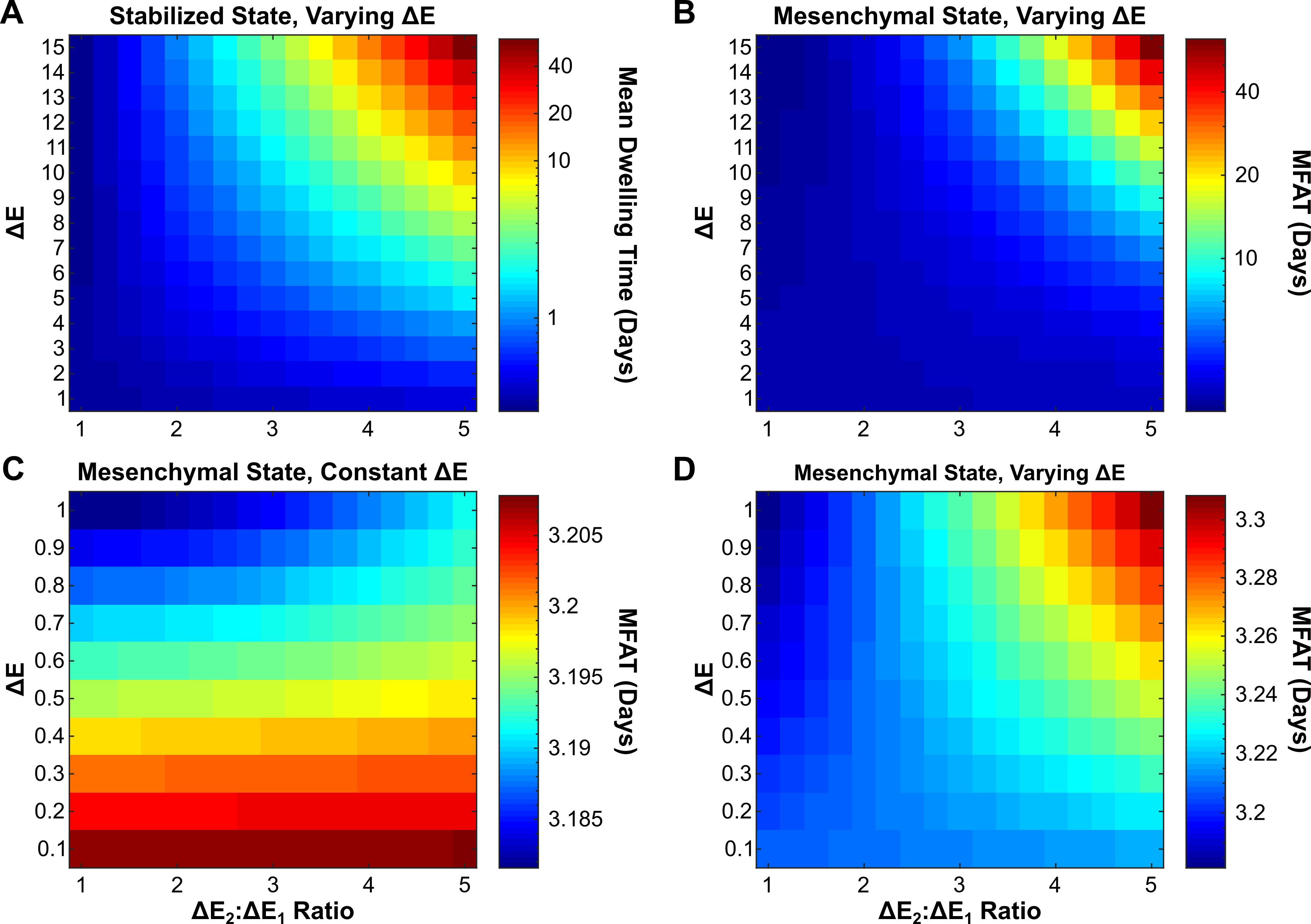

Supplement: S7 Fig — (A-B) Phase diagram of the mean dwelling time of the stabilized state (A) and the MFAT to the mesenchymal state (B) on the energy barrier ratio, ΔE2: ΔE1, and total energy barrier, ΔE, for the varying ΔE case. (C-D) Phase diagram of the MFAT to the mesenchymal state on energy barrier ratio ΔE2: ΔE1 and total energy barrier ΔE (≤ 1) in the constant ΔE case (C) and the varying ΔE case (D). (TIFF) [file pcbi.1007682.s007.tiff]
